# Recent Advances in Endodontic Diagnosis and Modern Treatment Plans

**DOI:** 10.3390/diagnostics13172786

**Published:** 2023-08-29

**Authors:** Alfredo Iandolo

**Affiliations:** Department of Medicine and Surgery, University of Salerno, 84081 Salerno, Italy; aiandolo@unisa.it; Tel.: +39-3287028233

Generally, to achieve success in endodontics, it is essential to perform all stages of treatment with cautiousness and excellence [1,2].

The first stage, one of the most important, is the diagnosis. At this stage, only with the help of modern technologies is it possible to carefully examine the fundamental problems of the tooth and organise a correct treatment plan [3,4]. For example, by making an accurate diagnosis, to know in advance whether that tooth can be saved or needs to be extracted. This decision is vital because a suitable treatment will be organised, preventing the patient from unnecessarily spending time and money.

Among the fundamental equipment for an effective diagnosis, we have three-dimensional radiology, the 3D CBCT [5].

Moreover, utilising this equipment, we can achieve a series of advantages; for instance, precise analysis of internal and external resorptions, detection of pathologies of the maxillary sinus of odontogenic origin, visualisation of root morphology, observation of small lateral or periapical lesions, precise control of healings, identification of fractures [6,7]. All of the above mentioned is impossible to accomplish using traditional 2D radiology.

Furthermore, modern 3D CBCT equipment can carry out targeted scans of small dimensions, only on the tooth or a few teeth to be analysed. Consequently, the radiation doses decrease, avoiding exposing the patient to excessive doses. In addition to radiology, in the diagnostic phase, it is essential to analyse the vitality of the teeth, any cracks, the periodontal status, etc. [8,9,10].

Only after having performed a correct diagnosis is it practicable to proceed with the subsequent stages of endodontic treatment: isolation of the operating field, access cavity, mechanical shaping, chemical cleaning, obturation, and finally, a suitable restoration [11,12,13,14,15].

Recent approaches and devices are available that allow a general dentist or a recent graduate to have safe and reproducible treatments.

The latest generation of rotating files is highly flexible and simple; it comprises effective cleaning techniques and modern obturation techniques that use the new biosealers [16,17,18,19,20,21,22,23,24,25].

In conclusion, each stage should be performed excellently using modern technologies and protocols to achieve success.
